# Role of TRPC6 in periodontal tissue reconstruction mediated by appropriate stress

**DOI:** 10.1186/s13287-022-03055-z

**Published:** 2022-08-05

**Authors:** Li Wang, Hong Liang, Bingjing Sun, Jing Mi, Xianqin Tong, Yuhui Wang, Meihua Chen, Liming Yu, Jie Pan, Shangfeng Liu, Yan-Jun Liu, Yuehua Liu

**Affiliations:** 1 Department of Orthodontics, Shanghai Stomatological Hospital, Fudan University, Shanghai, China; 2 Shanghai Key Laboratory of Craniomaxillofacial Development and Diseases, Shanghai Stomatological Hospital, Fudan University, Shanghai, 200001 China; 3 Dental Department, Shanghai 1st People’s Hospital Affiliated to Shanghai Jiao Tong University, Shanghai, China; 4 Shanghai Key Laboratory of Medical Epigenetics, International Co-Laboratory of Medical Epigenetics and Metabolism (Ministry of Science and Technology), Institutes of Biomedical Sciences, Department of Systems Biology for Medicine, Fudan University, Shanghai, 200032 China

**Keywords:** TRPC6, Mechanical force, Periodontal ligament stem cells, *TRPC6*^−/−^ mice, A confined microenvironment

## Abstract

**Introduction:**

The basis of orthodontic tooth movement (OTM) is the reconstruction of periodontal tissue under stress. Increasing the speed of OTM has always been the focus of attention.

**Objectives:**

Periodontal ligament stem cells (PDLSCs) are direct effector cells of mechanical force, but the mechanism by which PDLSCs sense mechanical stimuli is unclear.

**Methods:**

Human PDLSCs (hPDLSCs) were analyzed in the presence or absence of force loading with the Flexcell loading system in vitro. Then, periodontal tissues were analyzed after mechanical stimulation in vivo. In addition, cells in a confined microenvironment were analyzed to observe changes in the cytoskeleton and migration. Finally, TRPC6^−/−^ mice were used to further verify the effect of TRPC6. After force application, the OTM distance, bone marrow density (BMD), TRPC6 and COL1 expression, and TRAP staining were evaluated in periodontal tissues.

**Results:**

RNA sequencing (RNA-seq) and western blot analyses revealed that TRPC6 was important during mechanical force application to hPDLSCs. Appropriate mechanical force application also induced TRPC6 activation in the OTM model and the confined microenvironment. Under a slightly confined microenvironment, treatment with the TRPC6 inhibitor SKF96365 and TRPC6 knockout decreased the migration speed of hPDLSCs and mouse bone marrow mesenchymal stem cells (mBMSCs). In addition, TRPC6^−/−^ mice showed lower OTM distances and reduced osteogenic and osteoclastic differentiation.

**Conclusion:**

In summary, TRPC6 activation in PDLSCs mediated by appropriate mechanical force application contributes to periodontal tissue reconstruction.

**Graphical abstract:**

PDLSCs modulate periodontal tissue remodeling under appropriate mechanical stimulation through TRPC6; however, under excessive stress, alveolar bone and tooth roots are readily absorbed. Under this condition, environmental factors play a leading role, and the regulatory effect of TRPC6 is not obvious.
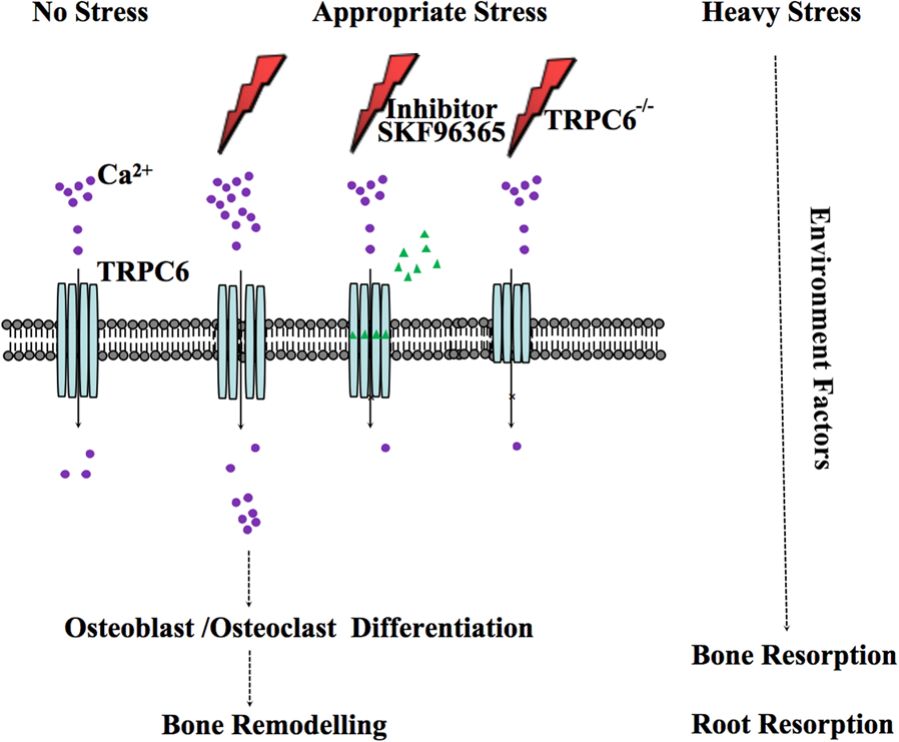

**Supplementary Information:**

The online version contains supplementary material available at 10.1186/s13287-022-03055-z.

## Introduction

With the gradual increase in people’s awareness of oral health care and aesthetics, orthodontic treatments aiming at health, beauty and orthodontic stability have become a common choice. However, perfect orthodontic treatment is often a long and complex process that usually takes more than two years. Therefore, identifying approaches to improve the speed of OTM has always been the focus of attention. To accelerate OTM, it is important to research the mechanism of periodontal tissue reconstruction under mechanical force.

Mechanical force plays an important role in tissue development and reconstruction and also in OTM [1–4]. Excessive mechanical force leads to the absorption of alveolar bone and roots, and insufficient force cannot generate the movement of teeth. Therefore, the application of appropriate orthodontic force is very important. The basis of OTM is the reconstruction of periodontal tissue under stress.

In the process of OTM, PDLSCs are the direct effector cells of mechanical force [5]. They sense mechanical stimulation and then translate these signals into biochemical signals to complete the process of periodontal tissue reconstruction [6, 7]. However, the mechanism by which PDLSCs sense mechanical stimulation remains unclear and needs to be further studied.

Mechanosensitive ion channels (MICs) are the main mechanoreceptors on the surface of living cells when a mechanical stimulus is transduced into an intracellular electrical or chemical signal. MICs form a mechanoreceptor system together with the cytoskeleton [8]. MICs mainly include epithelial sodium channels (ENaCs), two-pore potassium channels (K2Ps), transient receptor potential channels (TRPs) and piezo electric ion channels (Piezos).

Transient receptor potential (TRP) calcium channels are mechanosensitive channels and can sense different stimuli [9, 10]. Transient receptor potential channel 6 (TRPC6) is a nonselective cation channel that plays an important role in regulating calcium influx in most nonexcitatory cells [11]. Previous studies have shown that TRPC6 is widely expressed in the brain, kidney, smooth muscle tissue, immune cells and blood cells and is mechanosensitive [12–14]. Our previous study showed that TRPC6 played a significant role in the osteogenic differentiation of PDLSCs [15], but the role of TRPC6 in PDLSCs under stress was unclear. Therefore, we hypothesized that PDLSCs are affected by mechanical stimulation, which induces the activation of TRPC6 and further promotes periodontal tissue reconstruction.

Here, using a tensile and compressive force stimulus in vitro and an OTM model in vivo, we assessed the regulatory effect of TRPC6 under stress. In addition, the cells in a confined microenvironment were used to further verify the effect of TRPC6. *TRPC6* knockout mice have been used in the pulmonary vasculature [16], so we also used *TRPC6*^−/−^ mice to further verify the effect of TRPC6 during bone remodeling after appropriate mechanical force application. In conclusion, we show that the activation of TRPC6 in PDLSCs under appropriate stress is helpful to regulate bone remodeling during tooth movement, to provide new methods and ideas for accelerating orthodontic tooth movement, which has clinical guiding significance.

## Material and methods

### Ethics statement

The Ethics Committee of Shanghai Stomatological Hospital (China) has granted the study protocol, and informed consent was got from the parents of three donors (one 12-year-old female and two 13-year-old males) following the Declaration of Helsinki.

### Cell culture and treatment

HPDLSCs were removed from the root surface of healthy premolars extracted following orthodontic extraction. Culture methods were applied as reported in the literature [15]. Cells between passages 4 and 8 were used for experiments.

Mouse bone marrow mesenchymal stem cells (mBMSCs) were obtained from the tibias and femurs of 4-week-old wild-type mice and *TRPC6*^−/−^ mice. Culture methods were applied as reported in the literature [17]. When mBMSCs were 80% confluent, they were passaged for all experiments.

### Cyclic strain experiments

HPDLSCs were cultured on six-well Bioflex plates (Flexcell International, Hillsborough, NC, USA) at a density of 3*10^5^ cells/well. At 90% confluence, cells were serum-starved in α-MEM for 24 h and were then subjected to cyclic strain using a Flexcell Tension Plus system (FX-5000 T, Flexcell International, USA) (10%, 0.1 Hz) for 6 or 24 h. As static controls, cells were seeded into the same kind of plates but were not subjected to cyclic strain.

### 3D scaffold preparation

Cells at a density of 4*10^7^ cells/ml were mixed with an equal volume of 4% low melting point agarose (Sigma, Merck KGaA, Darmstadt, Germany). The mixture was added to 6-well BiopressTM compression culture plates (Flexcell International, Burlington, NC, USA) and allowed to solidify at room temperature.

### Cyclic compression experiment

Cells within the 3D scaffolds were incubated in α-MEM for 24 h and then subjected to cyclic strain using a Flexcell-5000C™ compression system (Flexcell International, Burlington, NC, USA) (2 kPa, 0.1 Hz) for 6 and 24 h [18–21]. Unloaded 3D scaffold samples were used as static controls.

### Fabrication of confinement slide with different height

The new silicon wafer (Yangzhou Genesis Microelectronics Co., Ltd, China) was washed with isopropanol and dried with gentle nitrogen. And then silicon wafer was placed on a heating plate at 120 °C for 30 min. After recovery to room temperature, the silicon wafer was coated with an SU-8 photoresist according to the instructions to obtain different heights by a spin coater (SPIN200-IND, Germany). And the soft-baked temperature was set up following the SU-8 photoresist instructions. The prepared silicon was covered by a patterned mask which was designed before this experiment and exposed under the mask aligner (URE-2000S/A, China). Sequentially, the silicon wafer was immersed in the developer completely. After rinsing the silicon wafer with isopropanol to remove the developer completely, the silicon was prepared for fabricating different height confinement slides (3 μm, 5 μm, 8 μm, 10 μm). The height of the silicon wafer was measured by an optical profilometer (Alphastep D-600). The glass slides (diameter, 12 mm, Matsunami, Japan) were cleaned with plasma cleaner (Harrick PDC-002, USA) for 3 min before using. The silicon wafer was covered with RTV615 PDMS (Momentive, USA) mixture (A/B, 8/1, w/w), and then the glass slide was gently pressed on PDMS. After heating at 120 °C for 15 min, the confinement slide containing PDMS pillars was prepared.

### Construction of cell confined microenvironment in vitro

The experiment methods were applied according to the reported literature [22]. Briefly, the large PDMS pillars (A/B, 35/1, w/w) were stuck on the lid of the 6-well glass bottom plate (Matsunami, Japan), which were made before using the custom mold. The PDMS pillars on the glass slide were modified with 0.1 mg/mL PLL(20)-g [3.5]-PEG(2) (10 mM HEPES, pH 8.5) (SuSoS, Switzerland) at room temperature for at least 3 h. After rinsing with PBS (10 mM, pH 7.4), the glass slides containing PDMS pillars were stuck on the other side of the large PDMS pillars.

The hPDLSCs or mBMSCs were seeded into a 6-well glass bottom plate one day in advance. The cells were stained with SiR-actin (200 nM) for 6 h and Hoechst (200 ng/mL) for 1 h at least. To inhibit the activation of *TRPC6*, SKF96365 (100 µM) (Selleck, USA) was added to the medium for 1 h before incubation in the confined microenvironment [23]. The prepared lid of the 6-well glass bottom plate was vertically covered on the cells and maintained with adhesive tape (Scotch Magic Tape, 3 M).

### Live-cell imaging

The motility of live-cell under confinement microenvironments was acquired by an inverted fluorescence microscope with 20 × objective (Leica, Germany). All cells were stained using Hoechst (cell nucleus) and SiR-actin (cell actin cytoskeleton). The microscope was equipped with a humidified incubation chamber at 37 °C and 5% CO_2_ at all times. MetaMorph software (Universal Imaging) was used to analyze cell migration images.

### Image analysis

Cells trajectories were tracked from fluorescence images of Hoechst staining with ImageJ (using the TrackMate or manual tracking with the TrackMate plugin) and a home-written Matlab program. We calculated cells speed according to the reported literature [22].

Cell cytoskeleton analyses were obtained from fluorescence images of SiR-actin and analyzed by ImageJ software.

### Construction of transcriptome libraries and sequencing

Total RNA was extracted from hPDLSCs after application of tensile force and pressure force in vitro using TRI reagent (Sigma-Aldrich, Germany). RNA-seq libraries were generated using these RNA sequences by RNA fragmentation, cDNA synthesis with random hexamer primers, linker ligation and amplification. Illumina HiSeq 2000 platform was used to sequence each library.

### RNA-seq data analysis

For gene expression annotation, the RPKM (reads per kb per million mapped reads) value of each gene was counted, and DEGs were defined as those with a fold change of ≥ 2.5 and a *P* value of ≤ 0.01. For analysis of expression patterns, hierarchical clustering, functional enrichment and protein–protein interaction analyses were conducted with the omicsbean website (http://www.omicsbean.cn) using the DAVID Gene Ontology database.

### Quantitative reverse transcription-polymerase chain reaction (PCR)

Total RNA was extracted from hPDLSCs in the confined microenvironment and from mBMSCs isolated from wild-type mice and *TRPC6*^−/−^ mice, and reverse transcription and real-time PCR were performed as reported in the literature [24]. The primers were designed using Primer Premier 5.0 software and are listed in Table 1.

**Table 1:** Primers used for real-time polymerase chain reaction

Gene	Forward primer (5′ → 3′)	Reverse primer (5′ → 3′)
*hGAPDH*	AAAATCAAGTGGGGCGATGC	TGGTTCACACCCATGACGAA
*hTRPC6*	GTGATCGCTCCACAAGCCTAT	CTGCCAACTGTAGGGCATTCT
*mβ-actin*	GCCCTGAGGCTCTTTTCCAG	TGCCACAGGATTCCATACCC
*mTRPC6*	ATCTGCTCATGGACTCGGAG	AACCTTCTCCCCTTCTCACG

### Western blot analysis and antibodies

HPDLSCs after application of tensile force, pressure force were scraped with ice-cold RIPA buffer (Thermo Fisher Scientific, USA). The method of western blot was applied according to the reported literature [15]. Membranes were incubated overnight at 4 ℃ with the following primary antibodies: anti-TRPC4 (Sigma, MO); anti-TRPC6 (Abcam, UK) and anti-β-actin (Sigma, MO). ImageJ software was used to analyze the relative density of each band.

### Animal models and orthodontic force application

All protocols were approved and conducted in accordance with the regulations of the Institutional Animal Care and Use Committee of Shanghai Model Organisms Center (China). 24 Sprague–Dawley rats and 48 male mice (24 wild-type mice and 24 *TRPC6*^−/−^ mice) were used. *TRPC6*^−/−^ mice were constructed at the Shanghai Model Organisms Center using *CRISPR/Cas9* genome editing (Additional file 1: Fig. S1) [15].

To establish the OTM model in rats, 24 Sprague–Dawley rats (male, 8 weeks old) weighing 200 g were used: 6 rats per day on Days 1, 3, 7 and 14, with at least two rats per day for micro-CT and immunohistochemical staining. To generate tooth movement, a nickel-titanium coil spring (Smart Technology, China) was used to connect the maxillary right first molar to the incisors (actuation force = 60 g). The left first molar was used as the control.

To establish the OTM model in mice, 24 wild-type mice and 24 *TRPC6*^−/−^ mice (male, 4 weeks old) weighing 20 g were used: 6 mice per day on Days 1, 3, 7 and 10, with at least two mice per day for micro-CT and immunohistochemical staining. The method of appropriate mechanical force application was as described above, but the actuation force was 20 g.

### OTM distance measurement and micro-CT analysis

Using an overdose of pentobarbital sodium, we sacrificed the rats and mice and harvested the maxillae. The OTM distance refers to the distance between the first molar and the second molar. Then, we used a high-resolution micro-CT system (Scanco Medical, Switzerland) to image the maxillae. We chose the furcation of the first molar as the location for analysis of bone mineral density.

### Immunohistochemical staining

Immunohistochemical staining was conducted as previously described [25]. Anti-TRPC6 (ab62461; Abcam, Cambridge, UK) and anti-COL1 (ab34710; Abcam, Cambridge, UK) primary antibodies were used.

### TRAP staining

We performed TRAP staining with an acid phosphatase kit (Sigma, USA) according to the manufacturer’s instructions as previously described [25]. We chose 3–5 randomly selected fields near the PDL for counting of TRAP-positive cells.

### Statistical analysis

The experimental data are shown as the means ± standard deviations, and each experiment was repeated three times independently. Statistical analyses and mapping were performed with GraphPad Prism 8. For multiple groups of samples, if the data obeyed the normal distribution and the variance was homogeneous, ANOVA was used, and the Bonferroni test was used for pairwise comparisons; if the data obeyed the normal distribution but the variance was nonhomogeneous, Welch test was used, and Dunnett’s T3 test was used for pairwise comparisons; if the data were nonnormally distributed, the Kruskal–Wallis test with the *X*^2^ statistic was adopted, and the Bonferroni test was used for pairwise comparisons. *P* < 0.05 was considered to indicate a significant difference.

## Results

### The rapid identification of the important stress-sensing gene TRPC6 by RNA-seq

**Fig. 1:**
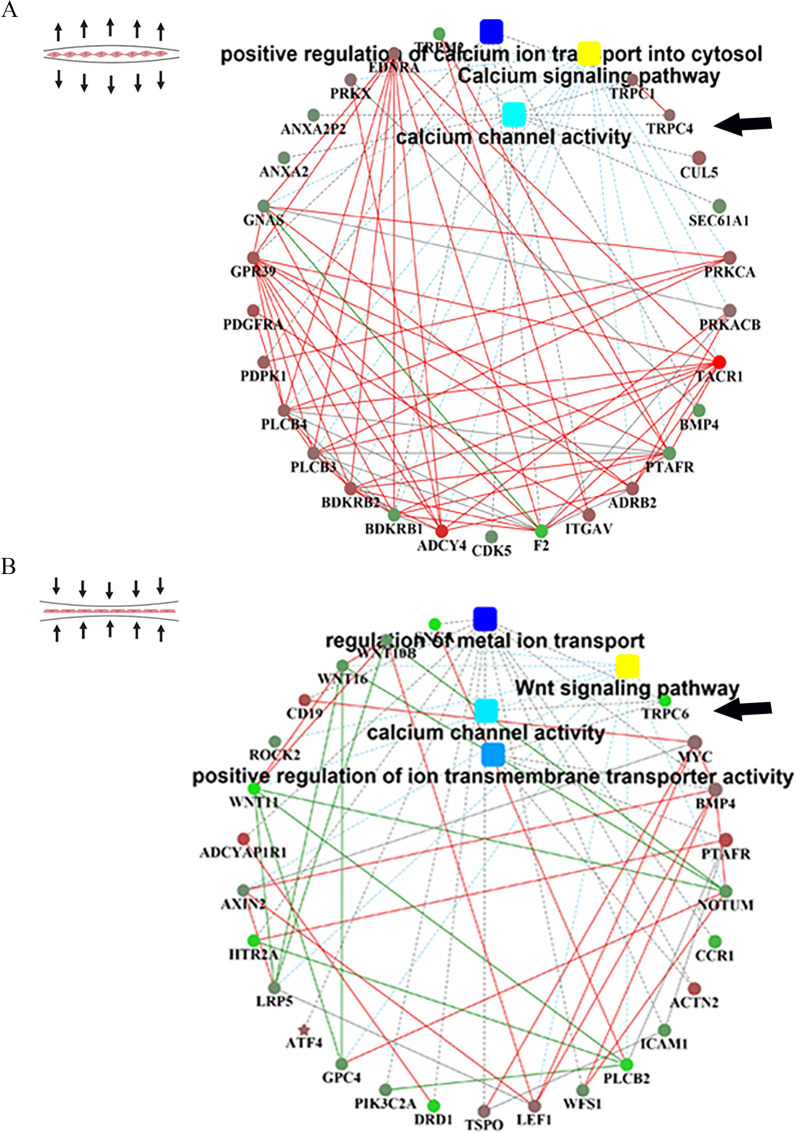
Analysis of the DEGs and interaction networks under stress. **A** Cell tension pattern and interaction network of the DEGs under tension at 24 h. **B** Cell compression pattern and interaction network of the DEGs under compression at 6 h

We investigated hPDLSCs after the application of tensile and compressive forces in vitro. Principal component analysis (PCA) was performed after the application of the tensile and compressive forces (Additional file 1: Fig. S2A, B). We identified 7091, 7722, 3302 and 6433 differentially expressed genes(DEGs) in the cells compared with the control cells after tensile force application for 6 h, tensile force application for 24 h, compressive force application for 6 h and compressive force application for 24 h, respectively. In the tensile force groups, we identified 4038 significantly upregulated DEGs and 3053 significantly downregulated DEGs between 6 h and control groups, as well as 3855 significantly upregulated DEGs and 3867 significantly downregulated DEGs between the 24 h and control groups (false discovery rate (FDR) < 5%, > twofold change). In the compressive force groups, we identified 1508 significantly upregulated DEGs and 1794 significantly downregulated DEGs between 6 h and control groups, as well as 2832 significantly upregulated DEGs and 3601 significantly downregulated DEGs between the 24 h and control groups (false discovery rate (FDR) < 5%, > twofold change), which formed distinct clusters (Additional file 1: Fig. S2C, D). The relative differential expression values for the different times are directly indicated on the heatmap (Additional file 1: Fig. S2E, F). According to PPI network analysis, we found several significant DEGs, such as *TRPC1, TRPC4, TRPC6, CUL5, PRKCA, BMP4, PLCB3, PLCB4, MYC, ROCK2, CD19, WNT16* and *Wnt11* (Fig. 1A, B). Due to our previous studies on calcium channels and mechanosensitive channels [26, 27], we focused on the TRPC channel, such as *TRPC4* and *TRPC6*.

### TRPC6 regulated the stress response of periodontal ligament stem cells

**Fig. 2:**
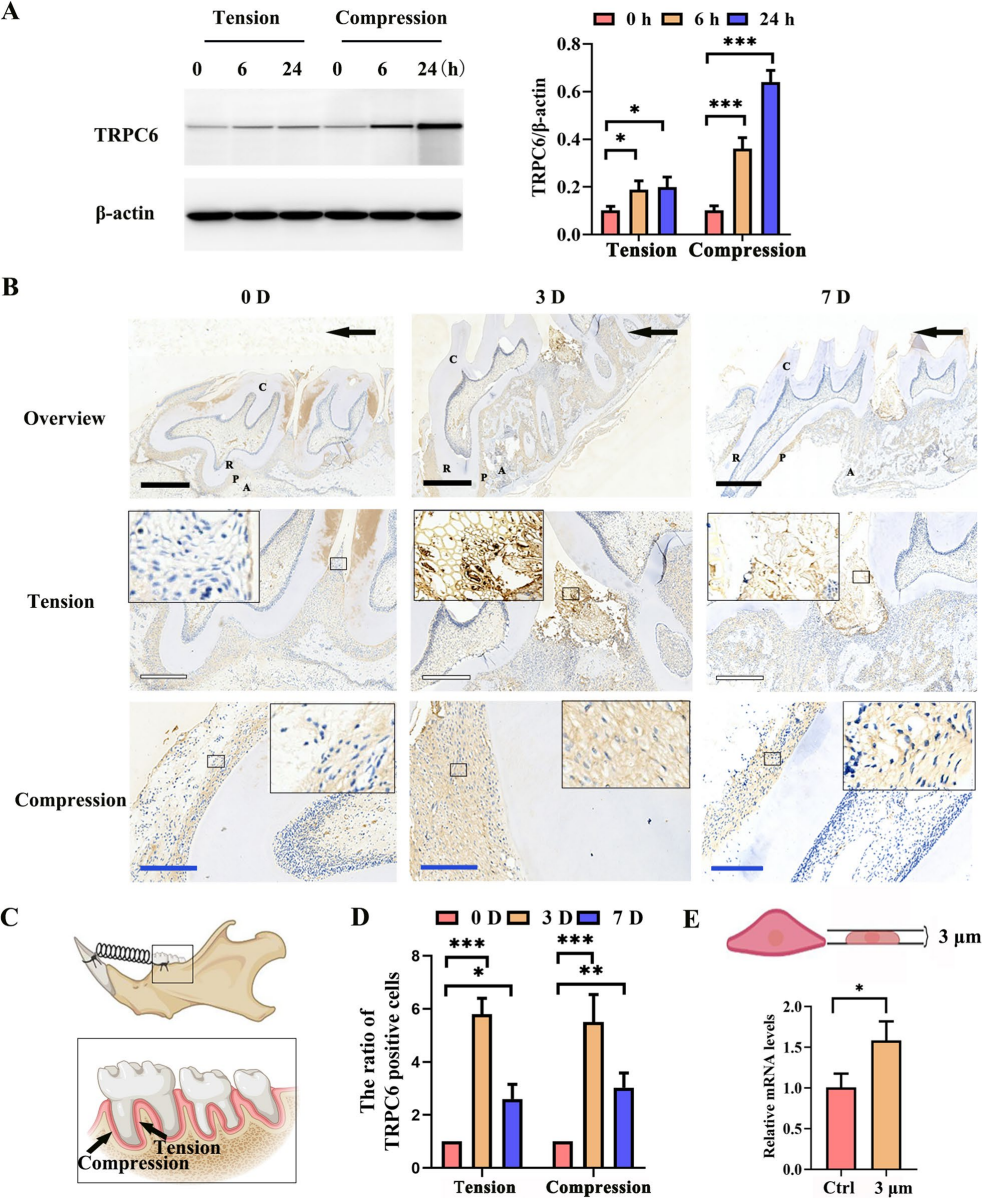
Mechanical force upregulated TRPC6 expression in PDLSCs. **A** The expression of TRPC6 as determined by western blotting and quantitative analysis of TRPC6 expression in hPDLSCs are shown. **B** The immunohistochemical images indicate that the expression of TRPC6 increased in molars subjected to orthodontic force. The black arrow indicates the direction of orthodontic force. C, crown; R, root; P, periodontal ligament; A, alveolar bone; Black scale bar, 1 mm; White scale bar, 500 μm; Blue scale bar, 200 μm. **C** Schematic diagrams. **D** Semiquantitative analysis of TRPC6-positive cells in immunohistochemical images. **E** The results of real-time PCR showed that the expression of *TRPC6* was significantly increased when hPDLSCs were exposed to a 3 μm confined microenvironment for 24 h. All the results are expressed as the mean ± standard deviation of three independent experiments. *P < 0.05; **P < 0.01; ***P < 0.001; ns, *P* > 0.05

To validate the RNA-seq results in vitro, we used western blotting to evaluate TRPC4 and TRPC6 protein expression in periodontal ligament stem cells after stress in vitro. HPDLSCs showed time-dependent activation of TRPC6 (Fig. 2A) and TRPC4 (Additional file 1: Fig. S3A) under both tensile and compressive stress. The change in TRPC6 was more obvious after compressive stimulation than after tensile stimulation. The change in the level of TRPC6 was greater than that in the level of TRPC4; thus, we mainly studied TRPC6.

In vivo, after force application, the proportions of TRPC6-positive cells on Days 3 and 7 were increased more than those in the control group (Fig. 2B-D). In addition, the proportions of TRPC4-positive cells on Days 3 and 7 were increased compared with those in the control group (Additional file 1: Fig. S3B). These results showed that TRPC4 and TRPC6, as sensors of mechanical force, were activated in PDLSCs after appropriate mechanical stimulation.

In addition, real-time PCR revealed that *TRPC6* expression was significantly increased in hPDLSCs in a 3 μm confined microenvironment (Fig. 2E), further verifying the mechanosensitivity of *TRPC6*.

### Inhibition of TRPC6 changed the morphology and migration speed of hPDLSCs in the confined microenvironment

We induced confinement by covering cells with an adhesive surface as previously described [21]. The initial height of the hPDLSCs was approximately 10 μm. At a height of 8 μm (slight restriction), the cell body was slightly deformed. At heights of 5 μm and 3 μm (high restriction), both the cell body and the nucleus were severely deformed [22, 28].

**Fig. 3:**
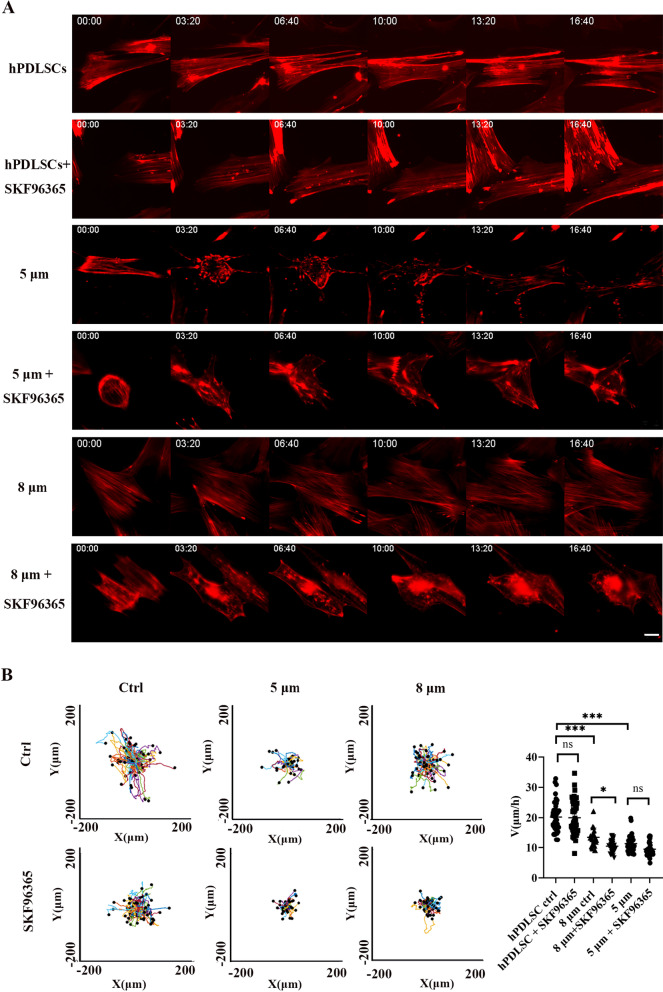
HPDLSCs in the 5 μm and 8 μm confined microenvironments. **A** In the 5 μm confined microenvironment, the actin bundles in the cytoskeleton disappeared, and the cell body became round and disordered. In the 8 μm confined microenvironment, the changes in the cytoskeleton were not obvious. Under treatment with the TRPC6 inhibitor SKF96365, the ability of cells to resist compressive stimulation decreased; thus, the cell morphology exhibited a relaxed state. Scale bar, 20 μm. **B** The cell migration trajectory decreased in the confined microenvironment. Environmental factors played a key regulatory role, and the role of TRPC6 was also significant in the 8 μm confined microenvironment. The results are expressed as the mean ± standard deviation of one independent experiment; *N* ≥ 3. *P < 0.05; ***P < 0.001; ns, *P* > 0.05

In the 5 μm confined microenvironment, the actin bundles in the cytoskeleton disappeared, and the cell body became round and disordered. Under treatment with the TRPC6 inhibitor SKF96365, the ability of cells to resist compressive stimulation decreased; thus, the cell morphology exhibited a relaxed state (Fig. 3A). In addition, we found that nuclear restriction (5 μm) decreased the speed of cell migration. Environmental factors played a key regulatory role, while the role of TRPC6 was not obvious (Fig. 3B).

In the 8 μm confined microenvironment, the changes in the cytoskeleton were not obvious. The cell migration trajectory decreased, while the TRPC6 inhibitor SKF96365 also decreased the speed of cell migration. Environmental factors played a regulatory role, while the role of TRPC6 was also significant (Fig. 3B).

### TRPC6 knockout changed the morphology and migration speed of mBMSCs in the confined microenvironment

MBMSCs were obtained from wild-type mice and *TRPC6* KO mice. The height of the mBMSCs was approximately 12 μm; thus, we chose to limit our investigation to heights of 5 μm and 10 μm. At a height of 10 μm (slight restriction), the cell body was slightly deformed. At a height of 5 μm (high restriction), both the cell body and the nucleus were severely deformed.

**Fig. 4:**
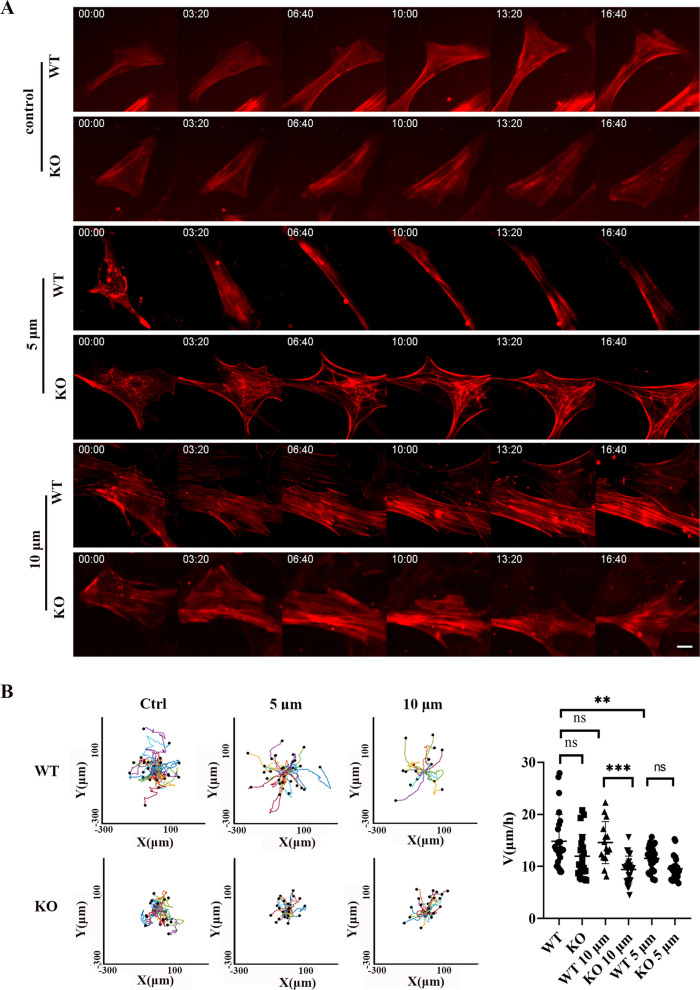
MBMSCs in the 5 μm and 10 μm confined microenvironments. **A** In the 5 μm confined microenvironment, mBMSCs resisted compressive stimulation, which made the cells acquire a rounded shape. In the 10 μm confined microenvironment, the changes in the cytoskeleton were not obvious. With *TRPC6* knockout, the ability of cells to resist compressive stimulation decreased; thus, the cell morphology exhibited a relaxed state. Scale bar, 20 μm. **B** The cell migration trajectory decreased with *TRPC6* knockout, and *TRPC6* knockout decreased the speed of cell migration in the 10 μm confined microenvironment. The results are expressed as the mean ± standard deviation of one independent experiment; *N* ≥ 2. **P < 0.01; ***P < 0.001; ns, *P* > 0.05

In the 5 μm confined microenvironment, mBMSCs resisted compressive stimulation, which made the cells acquire a rounded shape. In the 10 μm confined microenvironment, the changes in the cytoskeleton were not obvious. With *TRPC6* knockout, the ability of cells to resist compressive stimulation decreased; thus, the cell morphology exhibited a relaxed state (Fig. 4A), while the cell migration trajectory decreased, and TRPC6 knockout also decreased the speed of cell migration in the 10 μm confined microenvironment (Fig. 4B). Environmental factors did not play a regulatory role, while the role of *TRPC6* was significant. However, when the restriction was severe at 5 μm, environmental factors played a leading role, and the influence of the genetic factor *TRPC6* was not obvious (Fig. 4B).

### TRPC6 knockout suppressed osteoblast and osteoclast differentiation, leading to a shorter OTM distance

**Fig. 5:**
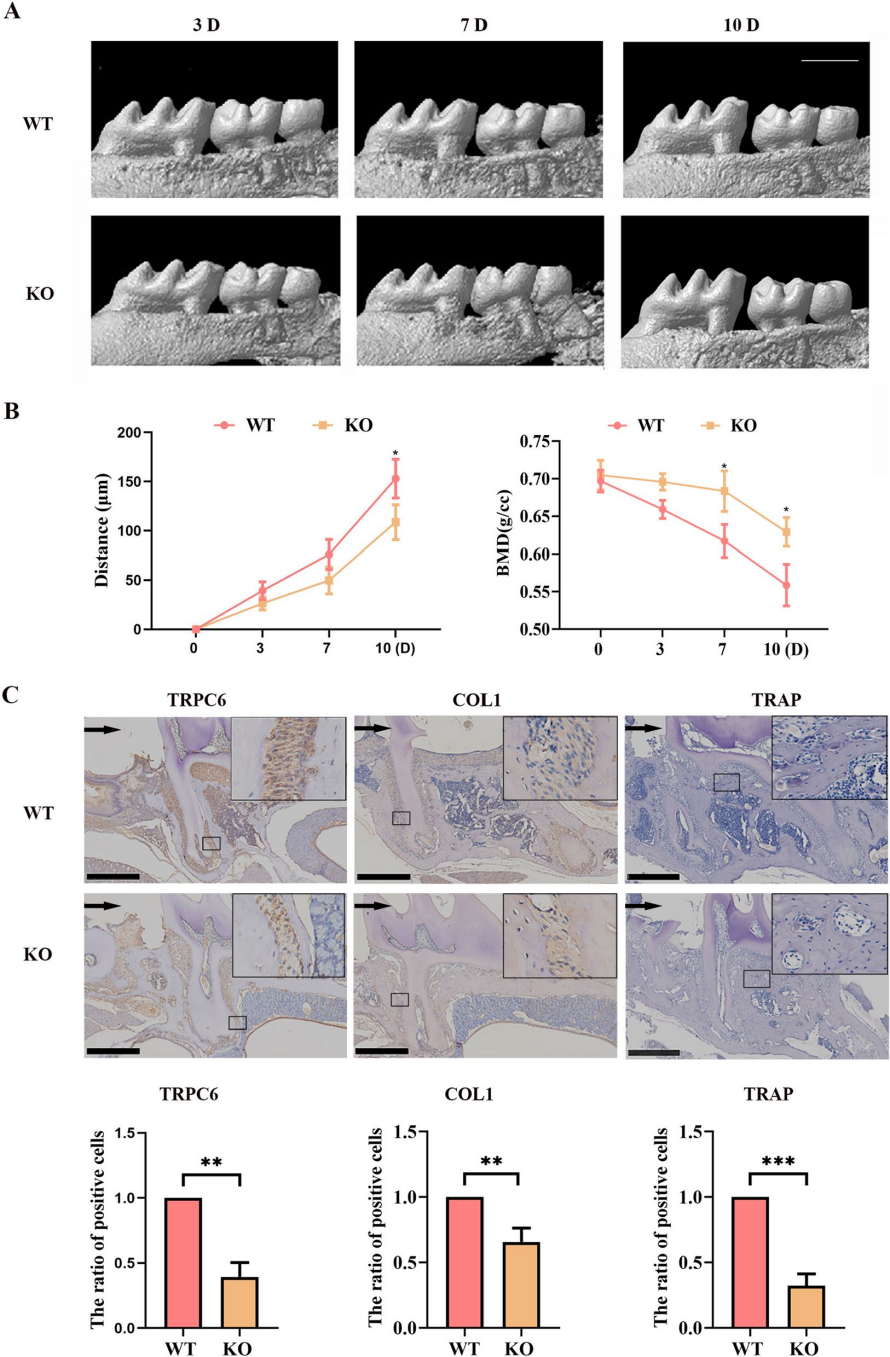
*TRPC6* knockout suppressed osteoblast and osteoclast differentiation, leading to a shorter OTM distance. **A** *TRPC6* knockout led to a shorter OTM distance. Scale bar, 1 mm. **B** *TRPC6* knockout led to slower bone mineral density conversion. **C** *TRPC6* knockout led to decreases in TRPC6 and COL1 expression and TRAP-positive osteoclasts. The black arrow indicates the direction of orthodontic force. Scale bar, 500 μm. Semiquantitative analysis of TRPC6 expression, COL1 expression and TRAP-positive osteoclasts. The results are expressed as the mean ± standard deviation of three independent experiments. *P < 0.05; **P < 0.01; ***P < 0.001

To test whether *TRPC6* plays a significant role in periodontal tissue remodeling, we used *TRPC6*^−/−^ mice (*TRPC6* KO mice) to prove the effect of *TRPC6*. We performed micro-CT scanning of the furcation region of the first maxillary molar, and we confirmed that *TRPC6* KO mice showed a decreased OTM distance and an increased BMD (indicating slower bone mineral density conversion) compared to those in wild-type mice (Fig. 5A, B).

On Day 7, histopathological examination of mice subjected to strain revealed that *TRPC6* KO mice showed lower TRPC6 staining and COL1 staining than wild-type mice (Fig. 5C). Consistent with this finding, we found a decrease in the percentage of TRAP-positive osteoclasts on the compression side of the alveolar bone in *TRPC6* KO mice compared to control mice (Fig. 5C). These in vivo findings collectively confirmed that the mechanosensitivity of TRPC6 is important in periodontal tissue remodeling.

## Discussion

TRPC6 is a nonselective cation channel. In our study, we showed that TRPC6 mediated the mechanical response of PDLSCs in an OTM model and cultured hPDLSCs subjected to tensile and compressive forces, contributing to periodontal tissue remodeling. First, the important stress-sensing gene *TRPC6* was rapidly identified by RNA-seq; second, upregulation of TRPC6 in PDLSCs was induced by appropriate mechanical force application both in vivo and in vitro; and third, the abovementioned hypothesis was further verified in confined microenvironments; finally, reductions in osteogenic and osteoclastic activities and tooth movement distances were observed in the OTM model of *TRPC6* knockout mice.

PDLSCs are mechanosensitive and are responsive to mechanical stimulation, which endows them with an important role in periodontal tissue remodeling [29–32]. However, the mechanism by which PDLSCs are involved in mechanosensing in periodontal tissue is unclear. In-depth study of gene expression profiles is complex, and RNA sequencing is one of the best methods for this type of research at present. HPDLSCs in the presence or absence of force loading with the Flexcell loading system in vitro were analyzed. By RNA-seq, we found that *TRPC4* and *TRPC6* were important in the transduction of mechanical stimuli in PDLSCs. TRPCs can be classified into four subsets according to their amino acid similarity: TRPC1, TRPC2, TRPC3/6/7 and TRPC4/5 [33]. Despite their differences, both TRPC4 and TRPC6 have been implicated as channels that may be sensitive to stress. TRPC4 and TRPC6 are expressed in various types of smooth muscle cells [34, 35]. Static strain increases TRPC4 mRNA expression in myometrial smooth muscle, and *TRPC6* knockdown in cerebral arteries inhibits pressure-induced depolarization and contraction [36, 37]. Based on the changes in the levels of TRPC6 and TRPC4 and our previous research, we demonstrated the regulatory effect of mainly TRPC6 under stress in vivo and in vitro.

In the study of mechanical force, we should not only consider whether the force is applied by tension or compression but also pay attention to the value. The force on the cells should not be too large; otherwise, the cells detach and die. The force should also not be too small. If it is too small, the mechanical signal may not be activated. Therefore, the “optimum force value” has also become a focus of attention. For tension, it is generally considered that 10–15% is the appropriate value and that values greater than or equal to 20% are excessive [38]. For compression, it is generally considered that the maximum value is 5 kPa [18–21]. Therefore, the appropriate forces used in our study were a tensile force of 10% and a compressive force of 2 kPa. In vivo, application of the appropriate force does not lead to adverse effects such as root resorption and alveolar bone resorption. The best force value can result in a faster tooth movement speed. Therefore, an actuation force of 60 g was used in rats, and an actuation force of 20 g was used in mice in our study [39].

This study is the first to prove that *TRPC6* is a mechanosensitive gene in periodontal tissue, providing novel insight into mechanosignaling. The confined microenvironment is the highlight and innovation of this study. In the process of orthodontic tooth movement, cells on the pressure side are under pressure, and there is also mutual extrusion between the cells, resulting in a confined microenvironment of the cells. The in vivo model and the Flexcell system cannot be used to visually observe the changes in a single cell under stress, but the confined microenvironment generated by microfluidics combined with microscopy can be used to observe the dynamic changes in a single cell under stress at the micron level. Real-time imaging is not possible in the in vivo model and Flexcell system, but the confined microenvironment we generated can provide this ability. Mesenchymal stem cells experience mechanical restriction of the cell body and nucleus in the confined microenvironment, but the effect of this mechanical signal on the morphology, migration and function of mesenchymal cells is not clear. The changes induced in the cytoskeleton and migration of cells in the confined microenvironment are similar to those induced under pressure, as shown in the referenced studies [40–42]. Therefore, the change in the migration velocity of periodontal ligament stem cells in a confined microenvironment leads to a change in the tooth movement velocity under the same conditions produced by TRPC6 knockout.

Through the discovery of TRPC6 and the related regulatory mechanisms, we found that TRPC6 plays an important regulatory role under both stress and confined microenvironment conditions. We have not conducted a detailed study on the different regulatory mechanisms of TRPC6. We expect to find a unified and important molecular regulatory mechanism of TRPC6. Therefore, we generated a confined microenvironment and observed the changes in the cells in the confined microenvironment at the micron level to confirm the regulatory role of TRPC6.

Gene knockout studies can more effectively explain the roles of related genes in regulating the mechanism of periodontal tissue remodeling and provide more reliable evidence. Therefore, in this study, *CRISPR/Cas9* genome editing was used to introduce mutations via nonhomologous recombination repair, resulting in a reading frame shift and functional loss of the *TRPC6* gene. In our study, *TRPC6* knockout suppressed osteoblast and osteoclast differentiation, leading to a shorter OTM distance, verifying the mechanosensitivity of TRPC6.

The identity of TRPC6 is a mechanically controlled cation channel has been confirmed by many studies [13, 14]. However, we found that the function of TRPC6 may not be limited to acting as an ion channel; it may also function as a cell membrane receptor that can transmit mechanical signals. The specific signal transmission mechanism is unclear; however, TRPC6 expression may change in response to mechanical stimuli, and TRPC6 expression increases after application of appropriate stress.

## Conclusion

In conclusion, PDLSCs modulate periodontal tissue remodeling in response to appropriate mechanical stimulation through TRPC6; however, when the stress is excessive, alveolar bone and tooth roots are readily absorbed. Under this condition, environmental factors play a leading role, and the regulatory effect of TRPC6 is not obvious. Further studies on the specific mechanism of TRPC6, experiments on mechanically controlled Ca^2+^ influx and studies in selected knockout mice should be performed to prove the regulatory role of TRPC6 in alveolar bone remodeling. In addition, we should devote more attention to the clinical application of TRPC6 and the important roles of small molecule compounds such as TRPC6 inhibitors and activators.

## Supplementary Information

**Additional file 1: Fig. S1**. The generation principle and identification of *TRPC6* knockout mice. (A) The generation principle of *TRPC6* knockout mice. (B) The scheme for identifying the genotype of *TRPC6* knockout mice (C) The results of real-time PCR showed that the expression of *TRPC6* was knocked out in mBMSCs from *TRPC6* knockout mice. **Fig. S2**. PCA, cluster and heatmap analyses under stress conditions. (A) PCA under tension. (B) PCA under compression. (C) Cluster analysis under tension. (D) Cluster analysis under compression. (E) Heatmap analysis under tension. (F) Heatmap analysis under compression. **Fig. S3**. Mechanical force upregulated TRPC4 expression in periodontal ligament stem cells. (A) The expression of TRPC4 as determined by western blotting and quantitative analysis of TRPC6 expression in hPDLSCs are shown. (B) The expression of TRPC4 increased after orthodontic force application in rats; a representative image of TRPC4 immunohistochemical staining on the tension and compression sides of molars subjected to orthodontic force is shown. The black arrow indicates the direction of orthodontic force. Black scale bar, 1 mm; White scale bar, 500 μm; Blue scale bar, 200 μm; Semiquantitative analysis of TRPC4-positive cells. The results are expressed as the mean ± standard deviation of three independent experiments. *P < 0.05; ***P < 0.001.

## Data Availability

The datasets used and/or analysed during the current study are available from the corresponding author on reasonable request.

## Abbreviations

OTM: Orthodontic tooth movement
PDLSCs: Periodontal ligament stem cells
hPDLSCs: Human PDLSCs
BMD: Bone marrow density
RNA-seq: RNA sequencing
mBMSCs: Mouse bone marrow mesenchymal stem cells
MICs: Mechanosensitive ion channels
ENaCs: Epithelial sodium channels
K2Ps: Two-pore potassium channels
TRPs: Transient receptor potential channels
Piezos: Piezo electric ion channels
TRPC6: Transient receptor potential channel 6
PCA: Principal component analysis
DEGs: Differentially expressed genes
FDR: False discovery rate
