# Supplementary material for: Role of TRPC6 in periodontal tissue reconstruction mediated by appropriate stress
Source: Stem Cell Res Ther. 2022 Aug 5;13:401. doi: 10.1186/s13287-022-03055-z (PMC9354362; doi:10.1186/s13287-022-03055-z)
Supplement: Supplementary file 1 — Additional file 1: Fig. S1. The generation principle and identification of TRPC6 knockout mice. (A) The generation principle of TRPC6 knockout mice. (B) The scheme for identifying the genotype of TRPC6 knockout mice (C) The results of real-time PCR showed that the expression of TRPC6 was knocked out in mBMSCs from TRPC6 knockout mice. Fig. S2. PCA, cluster and heatmap analyses under stress conditions. (A) PCA under tension. (B) PCA under compression. (C) Cluster analysis under tension. (D) Cluster analysis under compression. (E) Heatmap analysis under tension. (F) Heatmap analysis under compression. Fig. S3. Mechanical force upregulated TRPC4 expression in periodontal ligament stem cells. (A) The expression of TRPC4 as determined by western blotting and quantitative analysis of TRPC6 expression in hPDLSCs are shown. (B) The expression of TRPC4 increased after orthodontic force application in rats; a representative image of TRPC4 immunohistochemical staining on the tension and compression sides of molars subjected to orthodontic force is shown. The black arrow indicates the direction of orthodontic force. Black scale bar, 1 mm; White scale bar, 500 μm; Blue scale bar, 200 μm; Semiquantitative analysis of TRPC4-positive cells. The results are expressed as the mean ± standard deviation of three independent experiments. *P < 0.05; ***P < 0.001. [file 13287_2022_3055_MOESM1_ESM.docx]

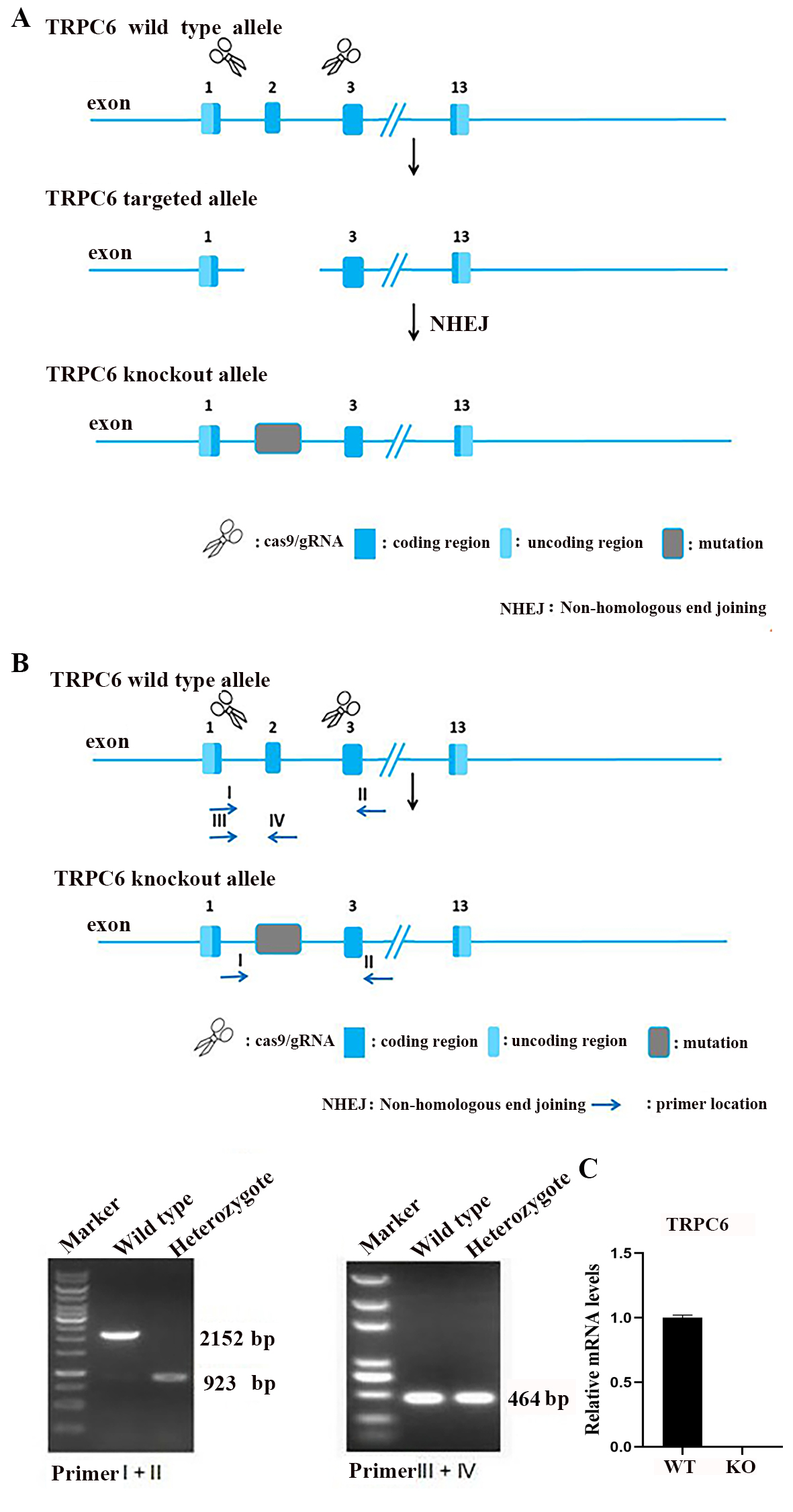


**Additional file 1: Figure S1**. The generation principle and identification of *TRPC6* knockout mice **(A)** The generation principle of *TRPC6* knockout mice **(B)** The scheme for identifying the genotype of *TRPC6* knockout mice (C) The results of real-time PCR showed that the expression of *TRPC6* was knocked out in mBMSCs from *TRPC6* knockout mice


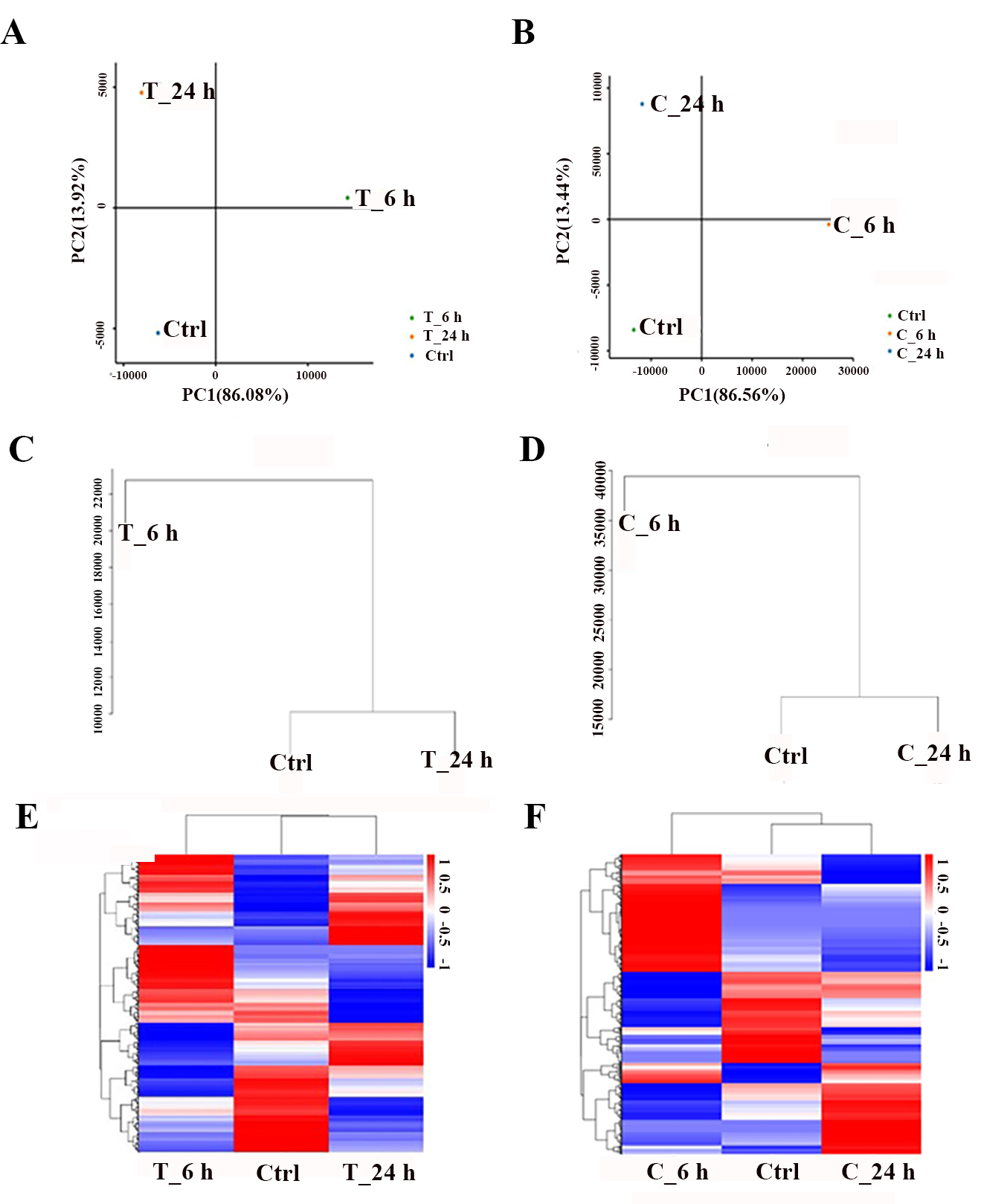


**Additional file 1: Figure S2**. PCA, cluster and heatmap analyses under stress conditions **(A)** PCA under tension **(B)** PCA under compression **(C)** Cluster analysis under tension **(D)** Cluster analysis under compression **(E)** Heatmap analysis under tension **(F)** Heatmap analysis under compression


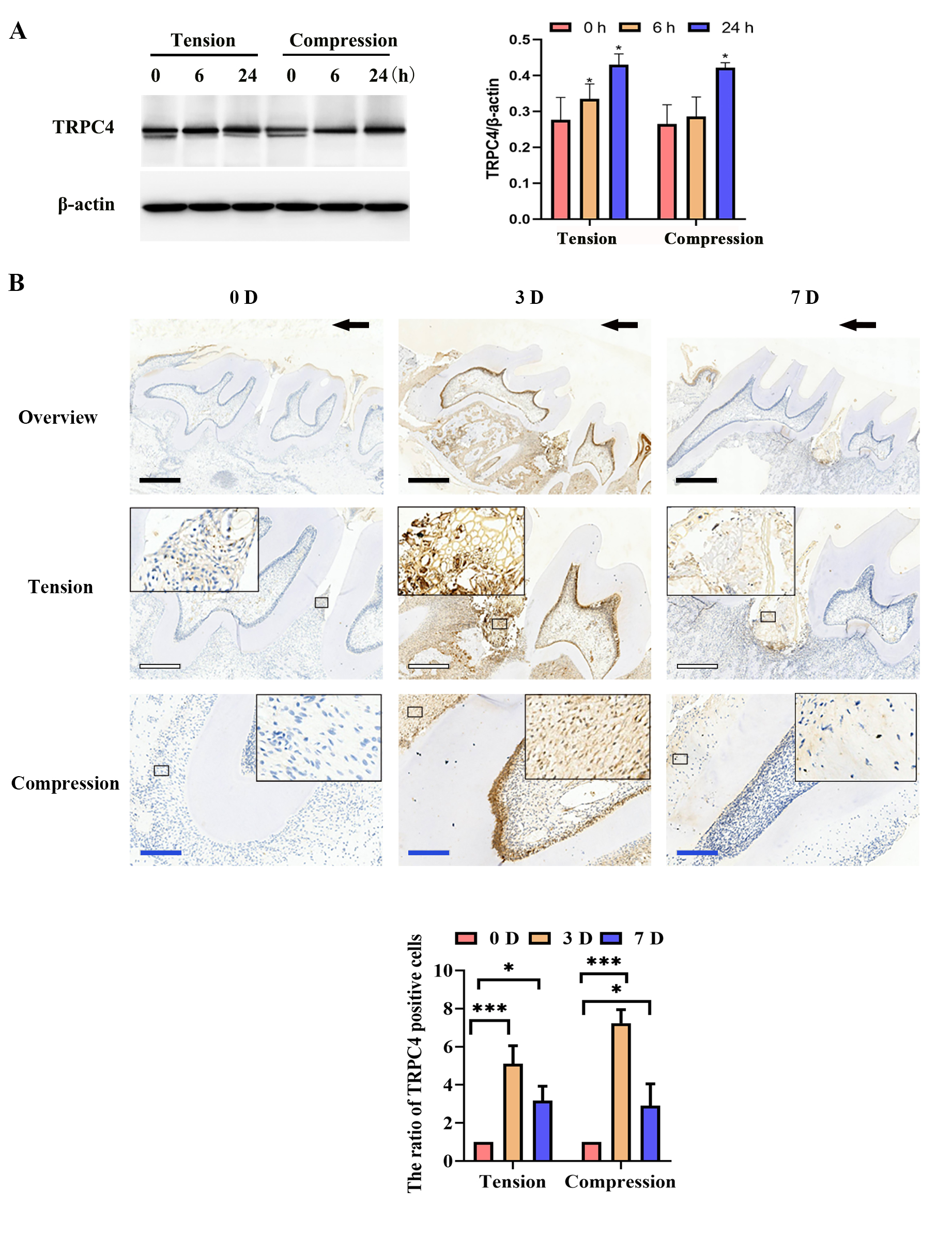


**Additional file 1: Figure S3**. Mechanical force upregulated TRPC4 expression in periodontal ligament stem cells **(A)** The expression of TRPC4 as determined by western blotting and quantitative analysis of TRPC6 expression in hPDLSCs are shown **(B)** The expression of TRPC4 increased after orthodontic force application in rats; a representative image of TRPC4 immunohistochemical staining on the tension and compression sides of molars subjected to orthodontic force is shown. The black arrow indicates the direction of orthodontic force. Black scale bar, 1 mm; White scale bar, 500 μm; Blue scale bar, 200 μm; Semiquantitative analysis of TRPC4-positive cells. The results are expressed as the mean ± standard deviation of three independent experiments. **P*<0.05; ****P*<0.001.
